# Transcatheter Treatment of Tricuspid Valve Disease: An Unmet Need? The Surgical Point of View

**DOI:** 10.3389/fcvm.2018.00098

**Published:** 2018-07-20

**Authors:** David C. Reineke, Eva Roost, Florian Schoenhoff, Miralem Pasic, Alex Kadner, Lars Englberger, Thierry P. Carrel

**Affiliations:** Department of Cardiovascular Surgery, University Hospital Bern, Bern, Switzerland

**Keywords:** tricuspid valve insufficiency, interventional therapy, surgery, new devices, future strategy

## Introduction

Tricuspid valve disease can either be stenotic or regurgitant. While stenotic lesions are very rare and mainly caused by rheumatic fever or carcinoid disease, the most common disease of the tricuspid valve is regurgitation (1). As with all valves, primary and secondary entities exist. This opinion paper will focus on the most frequent pathology, the functional tricuspid regurgitation (FTR), which primarily is considered as disease of the right ventricle.

## Why do we talk about the forgotten valve?

Most editorials on the tricuspid valve speak about “the forgotten or untreated valve.” Why is that so?

In our view, four facts are responsible for this development:

1. In the early seventies right sided endocarditis due to intravenous drug abuse was treated simply by surgically removing the valve, leaving behind torrential tricuspid regurgitation (TR). This was supported surprisingly well. Long-term data was of course scarce due to this selected patient population.
2. Initially it was believed that left-sided valve surgery would resolve the problem of the tricuspid valve. TR was generally thought to be a bystander with the main focus on the correction of left sided pathology. However it has been shown that TR does not resolve following left-sided heart surgery with relevant influence on long-term survival.
3. For a long time the focus was put on TR alone. Data has shown that not only the presence of TR represents a significant risk factor for severe TR during follow-up after mitral valve surgery, but that annular dilatation alone is a predictive factor for later TR.
4. In the pioneering days of heart surgery it was all about survival and TR was never demonstrated to adversely influence early outcome. This observation applied for the whole complex of right sided pathologies which only become relevant as patients' survival of left sided heart disease has considerably improved. A similar development can be seen in patients suffering from chronic pulmonary embolic disease. Right heart disease is just not as “appealing” as left heart disease.

## What do the guidelines tell us?

Guidelines supported this scientific and practical deficit on therapy for several decades. While American and European guidelines where relatively fast in listing a class I indication to perform tricuspid valve repair for severe TR in patients who are undergoing mitral valve (MV) surgery both guidelines where for a long period quite silent about performing concomitant tricuspid valve surgery for coronary bypass, aortic valve replacement or other cardiac surgery (2, 3).

The ESC Guidelines also listed tricuspid surgery as a Class I indication in the case of severe primary TR (or tricuspid stenosis) with symptoms and without severe RV dysfunction (3). The US guidelines on the other hand only listed this scenario as “reasonable” and as class IIa indication (2). This is one of the rare exceptions where valve disease with symptoms is not listed as a Class I indication for surgery in the guidelines (2) and reflects the general reluctance to operate on some of these patients who have already developed irreversible right ventricular (RV) dysfunction.

Patients with FTR remain asymptomatic for a long time despite considerably impaired right ventricular function (4). As a result the population with a potential indication to treat TR is of advanced age, has often already had cardiac surgery and suffers from extensive RV dysfunction. This is actually a shame as surgery performed in early stages of the disease is relatively easy and a straight forward procedure with very acceptable operative risk (5).

## Forgotten or just unnoticed?

Despite talk of the “forgotten valve,” already 20 years ago, renowned specialists in the field had the following messages which went unnoticed (5, 6):

- Tricuspid annular dilatation is more reliable than regurgitation and represents a more consistent landmark.
- TR alone is a unreliable parameter as it very much depends on several factors like volume status, preload, afterload and right ventricular dysfunction.
- TR is a continuing process, worsening despite treatment of left-sided pathologies. Already then the group around Gilles Dreyfus were able to show progression of the disease in 30% of patients during a follow-up between 5 and 15 years.

Treating annular dilatation beyond a given size improves functional status and there is a clear trend toward better survival compared to patients who did not receive treatment.

This concept is supported by several groups including the American College of Cardiology/American Heart Association guidelines who included a type IIa recommendation for patients with a threshold diameter of 40 mm (7–13).

The need of treatment of this polymorbid patient population has resulted in the development of a variety of interventional devices. But, it becomes evident that while we are again able to quite effectively treat left sided pathologies with TAVI and mitral devices, right-sided therapy options still stay far behind.

In the current ESC/ECTS guidelines with updated indications for the evaluation and surgical intervention in patients with TR, interventional strategies have only found their place in the chapter “Gaps in evidence”: “The potential role of transcatheter tricuspid valve treatment in high-risk patients needs to be determined (14).”

Sadly, the updated guidelines don't consider the tricuspid valve regurgitation as a general consequence of left sided heart disease but again have the tendency to stress the importance of the awareness of tricuspid disease in patients with significant mitral valve disease following data acquired after mitral clip therapy. In contrast, recent literature demonstrates that even after TAVI tricuspid valve dysfunction is associated with significantly increased mortality. This underlines that left-sided heart disease in general is responsible for FTR (15).

## Pathophysiologic background

From a morphological standpoint FTR is known for its dynamic disease progression and can be divided into three phases (16):

1. Dilatation of the RV results in dilatation of the tricuspid annulus. At this stage TR might not even be present depending on the degree of annular dilatation and lack of leaflet coaptation.
2. Progressive dilatation of the RV and tricuspid annulus will lead to severe lack of coaptation resulting in significant TR.
3. RV dilatation especially in the region of the free wall will in addition to annular dilatation lead to tethering of the tricuspid leaflets, due to the attachment of the papillary muscles of the tricuspid leaflets to the free wall of the RV. A tethering height of more than 8 mm is reported to be predictive of more than moderate functional TR.

## Surgical approaches

According to these three phases, surgical repair of the tricuspid valve for functional TR requires tailored strategies.

In the first two stages, tricuspid annuloplasty alone gives excellent results. The third phase however necessitates treatment of the annular dilatation as well as the leaflet tethering since annuloplasty alone is unlikely to be successful in treating TR. Here two options come into play—a reconstructive approach or the replacement of the valve.

A variety of supplemental techniques for addressing leaflet tethering, such as anterior leaflet patch augmentation, double- orifice valve or bicuspidization repair, have demonstrated efficient and lasting results. Not least, these techniques provide the concept and justification for many proposed catheter-interventional approaches. Regarding the replacement of the valve, a potential recurrence of TR due to disease progression is prevented (16). However, life-long and rather aggressive anticoagulation therapy after mechanical valve implantation or the inevitable long-term valve degeneration following implantation of a biological prosthesis present significant drawbacks. With the arrival of interventional valve-in-valve technology, the choice for a biological prosthesis appears to be preferable.

## Interventional approaches

Transcatheter tricuspid valve intervention (TTVI) is at an early stage of introduction into practice and only a few hundred patients have been treated so far (17, 18). The first interventional valve replacement was very recently performed (19).

According to their mode of action tricuspid valve catheter devices can be divided into four groups (20):

- Tricuspid valve annuloplasty devices
- Tricuspid edge-to-edge technique
- Heterotopic caval valve devices
- Coaptation devices

Due to the nature of the disease progression, the majority of patients qualifying for interventional treatment are usually those with advanced disease (phase two to three). According to extensive research done on surgically treated patients, most of the above mentioned interventional devices will not be effective in treating the pathology. It is our strong opinion, that industry and doctors should concentrate on annuloplasty devices and devices replacing the tricuspid valve. It would be unwise to flood the market with devices not able to treat the most common pathology and by that depriving very sick patients from an effective therapy.

## Conclusion

In conclusion, there is a clearly unmet need for the interventional treatment of TR mainly in very sick patients with FTR. Nonetheless device development and introduction must focus more on the mechanism of the disease in order to effectively treat it. The focus needs to be on annuloplasty devices and complete valve replacement strategies. One can only hope, that the current interest into interventional therapy of the tricuspid valve disease will bring back the “forgotten valve” into the conscience of the cardiological and surgical community. On the one hand, reminding cardiologists, that beside asymptomatic presentation of their patients a timely referral is mandatory for allowing a low-risk and optimal surgical intervention, on the other hand, reminding surgeons to address more consequently concomitant tricuspid valve disease and to apply repair strategies. The availability of interventional catheter-based techniques must not serve as an excuse for again “forgetting” the dysfunctional tricuspid valve and delaying therapy.

## Author contributions

DR: Idea and writing; ER: Correction and valuable Input; FS: Considerable Input; MP: Correction and Idea; AK: Discussion and Conclusion; LE: Final Correction; TC: Idea, Structuring, Correction.

### Conflict of interest statement

The authors declare that the research was conducted in the absence of any commercial or financial relationships that could be construed as a potential conflict of interest.
